# The Process of Maternal Recognition of and Response to Suspected Gaming Disorder Among Japanese Adolescents Receiving Psychiatric Outpatient Care: A Qualitative Study

**DOI:** 10.7759/cureus.116862

**Published:** 2026-09-24

**Authors:** Daisuke Takahara, Misuzu Takahara, Daisuke Nonaka

**Affiliations:** 1 Department of Global Health, Graduate School of Health Sciences, University of the Ryukyus, Ginowan, JPN; 2 Department of Mental Health Nursing, School of Health Sciences, Faculty of Medicine, University of the Ryukyus, Ginowan, JPN

**Keywords:** adolescents, gaming disorder, japan, mothers, psychiatric outpatient, qualitative research

## Abstract

Introduction: Gaming disorder (GD) is prevalent among adolescents, and those with neurodevelopmental conditions or symptoms of anxiety and depression may be particularly vulnerable to GD. Family-related factors, including the parent-child relationship, are also linked to adolescent gaming problems. However, less is known about how parents come to notice and respond to a child's gaming-related problems. This study aimed to explore, through mothers' retrospective accounts, how mothers of Japanese adolescents receiving psychiatric outpatient care who screened positive for suspected GD recognized and responded to gaming-related difficulties and associated smartphone use. The interviews also explored mothers' own experiences and the parent-child relationship in the course of responding to these difficulties.

Methods: This qualitative study used the Modified Grounded Theory Approach. Eligible participants were parents of a child aged 10 years or older who cohabited with them and engaged in internet gaming. The child was receiving outpatient psychiatric care. Data were collected between February and August 2024. Nine mothers whose children screened positive for suspected GD on the Gaming Disorder Scale for Parents were included in the analysis. Concepts were generated from interview transcripts and compared and refined through constant comparative analysis across cases to form categories.

Results: All nine children of participating mothers had at least one developmental or mental health diagnosis, and eight had two or more co-occurring diagnoses. Analysis generated 20 concepts, nine subcategories, and four categories. Mothers' accounts traced a process that began with the "necessity and personal ownership of smartphones," giving way to "uncontrolled game/smartphone use." As household rules went unenforced, mothers came to recognize increasingly serious problems, including "school non-attendance," "mental instability," and "trouble on social media," and mothers confronted these problems largely alone, reflected in "isolated child-rearing for mothers." Mothers also described experiences of "reaching professional support" and, in some families, "changes in parental awareness and behavior."

Conclusions: The process of maternal recognition and response involved isolation and fatigue, as mothers confronted problem behaviors, such as school non-attendance and emotional instability, largely on their own. These findings suggest that clinical assessment of families of adolescents with suspected GD may benefit from attending to mothers' own experiences alongside the child's symptoms.

## Introduction

Gaming disorder (GD), included in the 11th revision of the International Classification of Diseases (ICD-11), is characterized by impaired control over gaming, increasing priority given to gaming over other daily activities, and continuation of gaming despite negative consequences, resulting in significant functional impairment; these features are typically evident for at least 12 months, although a shorter duration may suffice if all diagnostic requirements are met and symptoms are severe [1]. A related construct, Internet gaming disorder (IGD), is described in Section III of the Diagnostic and Statistical Manual of Mental Disorders, Fifth Edition (DSM-5), as persistent and recurrent internet gaming associated with clinically significant impairment or distress, with a diagnosis requiring five or more of nine criteria to be present within a 12-month period [2]. GD encompasses gaming that may occur online or offline, while the DSM-5 construct of IGD specifically concerns internet gaming. Empirical comparisons of these two diagnostic frameworks suggest that the ICD-11 GD criteria place greater emphasis on functional impairment than the DSM-5 IGD criteria [3]. The pooled prevalence of IGD among adolescents has been estimated at 7.1% [4], while the prevalence of GD was estimated at 5.1% among Japanese youth aged 10-29 [5]. In addition, neurodevelopmental conditions such as autism spectrum disorder and attention-deficit/hyperactivity disorder have been associated with GD [6,7]. In a study of adolescents attending a Japanese psychiatric clinic, 15.3% of those with autism spectrum disorder and 30.3% of those with attention-deficit/hyperactivity disorder screened positive for gaming disorder using an ICD-11 screening tool [8]. IGD has also been associated with symptoms of anxiety and depression [9]. Beyond these clinical characteristics, Gao et al. reported the following possible risk factors for IGD among adolescents and young adults: stress, long average game time, family dysfunction, poor academic performance, being bullied, bullying, interpersonal problems, hyperactivity/inattention, anxiety, depression, emotional distress, and low self-esteem [10].

Systematic reviews link adolescent gaming problems to family-related factors such as parental mediation, family cohesion, and the quality of the parent-child relationship [11,12]. Lower symptom severity of IGD is associated with parental knowledge, active support, open communication, and age-appropriate monitoring, while greater severity is associated with parental hostility, including elevated conflict, rejection, and poor emotional regulation, as well as overcontrolling parenting [11]. One school-based study found a similar pattern: adolescents with problematic gaming behaviors had poorer family cohesion and worse parent-child relationships, and insufficient or maladaptive parental monitoring was a risk factor for both male and female adolescents [13]. Among Japanese male adolescents, overreactive parenting, marked by emotional outbursts and harsh discipline, has similarly been associated with suspected GD, whereas lax, inconsistent discipline has not [14]. Longitudinal evidence indicates that parental support predicts fewer future IGD symptoms, while parental abuse predicts symptom escalation, a finding that supports the case for parent-focused intervention [15]. IGD in adolescents also co-occurs with parental psychological distress, suggesting that assessing and supporting parents' own mental health may be an important part of caring for these families [16].

However, less is known about how parents come to notice and respond to a child's gaming-related problems. Qualitative studies centering on adolescents themselves exist, but those focusing on parents' own perspectives remain scarce; even when such studies exist, they have examined parents' perceptions only after their children had completed treatment [17], leaving the process of noticing and responding to the problem undescribed.

Given this gap, this study aimed to explore, through mothers' retrospective accounts, how mothers of Japanese adolescents receiving psychiatric outpatient care who screened positive for suspected GD recognized and responded to gaming-related difficulties and associated smartphone use. The interviews also explored mothers' own experiences and the parent-child relationship in the course of responding to these difficulties. By focusing on mothers' accounts, this study may help inform clinical support for families affected by adolescent GD in Japan.

## Materials and methods

Study design

This study employed a qualitative inductive design, in which semi-structured interview data were analyzed using the Modified Grounded Theory Approach (M-GTA) [18,19]. The phenomenon examined in this study, how parents recognize and make sense of their child's gaming- and smartphone-related problem behaviors, and how this recognition, together with their interactions with others, shapes their responses, has process-like characteristics in which perception, interaction, and response are interrelated. This research aim, to elucidate such a process of human action and interaction, was considered consistent with the characteristics of M-GTA.

Participants and data collection

The recruitment period for participants was February to August 2024. Eligible participants were parents of a child aged 10 years or older who cohabited with them and engaged in internet gaming. The child was an outpatient in the general psychiatric and developmental outpatient services of the study's collaborating clinical institution, a single specialized psychiatric hospital in Okinawa Prefecture, Japan. Parents who did not cohabit with a child meeting the age criterion, or whose child did not engage in internet gaming, were excluded. In-hospital poster advertisement was used for recruitment, and a convenience sample of parents who responded to the advertisement and met the eligibility criteria was enrolled. Data were collected through a single semi-structured, face-to-face interview per participant, conducted at the collaborating hospital by a co-investigator specializing in psychiatric nursing. This co-investigator had prior training and experience in qualitative interviewing, including previous published qualitative research, and had no prior clinical involvement in the care of the participating families or prior acquaintance with the participants. The duration of each interview was recorded. Interviews were audio-recorded with consent and transcribed verbatim. The interview guide covered background and demographic information about the parent and child, along with the child's gaming history (e.g., age at onset, household rules, and time spent gaming). The interview guide was developed collaboratively by the three co-investigators based on their clinical and research experience. No pilot study or formal external expert evaluation was conducted prior to its use. The interview included open-ended questions about the child's problem gaming behavior, the parent's own attitudes toward gaming, and concerns about the child's peer and family relationships. Interview questions served as a starting point for open-ended discussion rather than a fixed sequence, and the interviewer used follow-up questions and probes, generated in response to participants' responses, to clarify and elaborate on specific experiences and examples raised during the interview. Parents' recognition of and response to their child's gaming- and smartphone-related problem behaviors were elicited through several interview questions, including those addressing household rules and the use of parental monitoring functions (e.g., "Are there any family-established rules regarding gaming?"), and parents' concerns regarding problem behaviors, friendships, and the parent-child relationship (e.g., "What gaming-related problem behavior are you most concerned about?"), rather than through any single question addressing these processes directly. The full interview guide is provided in the Appendix. Each child's clinical diagnosis was obtained from medical records with the permission of the attending physician. Participants also completed a companion self-administered questionnaire, the Gaming Disorder Scale for Parents (GADIS-P), a validated screening measure for suspected GD based on the ICD-11 conceptualization of GD [20,21]. The GADIS-P comprises 10 items: nine symptom items, rated on a five-point Likert scale, that load onto two subscales, negative consequences (five items) and cognitive-behavioral symptoms (four items), and one additional item assessing the temporal criterion (frequency and duration of symptoms). Total scores on the nine symptom items range from 0 to 36, with higher scores indicating more severe GD symptoms. Their children were classified as screening-positive for suspected GD when three criteria were all met: a negative consequences subscale score of 6 or higher, a cognitive-behavioral symptoms subscale score of 10 or higher, and a score of 2 or higher on the temporal item (corresponding to symptoms having occurred for at least several days to one month, up to almost daily) during the past 12 months [20,21]. Permission to use the Japanese version of the GADIS-P was obtained from the developers. Only pairs meeting established GADIS-P screening-positive criteria were retained in the qualitative analytic sample, and their children were classified as having suspected GD.

Data analysis

M-GTA, developed by Kinoshita [18,19] as a systematic reorganization of Glaser and Strauss's [22] original grounded theory approach, is well established in Japan and has also been used in international health science research (e.g., Amano and Suzuki [23]). The analytic theme was established as "the process by which mothers of adolescents with suspected GD recognize and respond to their child's gaming- and smartphone-related problem behaviors", and the analysis focus person as "a mother who is the primary caregiver of an adolescent with suspected GD".

Analysis proceeded through the following steps: (1) in light of the analytic theme and analysis focus person, a relevant part of the data was identified and treated as an example, and a concept was generated that appeared to explain this and other similar examples as well; (2) in creating each concept, an analysis worksheet was created, recording the concept name, definition, and initial example; (3) as the analysis proceeded, new concepts were generated, with an analysis worksheet created for each concept; (4) in parallel, additional examples were sought from the data and added to the examples field of the worksheet; a concept was judged invalid if sufficient examples could not be found; (5) the completeness of each concept was assessed not only through similar examples but also by examining the data from the perspective of polar examples, guarding against the risk that interpretation would become arbitrarily biased; the results were recorded in the worksheet's theoretical notes field; (6) relationships between each generated concept and other concepts were then examined and depicted in a relationship diagram; (7) categories, formed from the relationships among multiple concepts, were generated, and the analytic findings were integrated based on the relationships among categories, summarized concisely in writing as a storyline, with a results diagram also created. Data were manually organized and analyzed using Microsoft Word and Excel (Redmond, WA, USA) to create and manage the analysis worksheets, consistent with the standard M-GTA procedure [18,19].

Trustworthiness

Credibility was addressed through mechanisms internal to M-GTA. The first author conducted the primary analysis and discussed the interpretation of the data with the co-investigators, who reviewed the analysis throughout the analytic process. When interpretations of specific concepts or their relationships differed among the researchers, these differences were discussed within the research team with reference to the analysis worksheets and the original transcripts until agreement was reached. Each concept was documented on an analysis worksheet with its definition and supporting examples, and concepts were refined through constant comparison across cases, with explicit attention to disconfirming instances. Consistent with the principles of M-GTA, theoretical saturation was judged not on the basis of the frequency or number of participants reporting a given concept, but on whether new concepts continued to emerge as the analysis proceeded and whether the emerging concepts and category relationships adequately reflected the range of experiences described by participants. This was assessed continuously throughout the analytic process by examining whether each subsequent transcript yielded concepts not already captured by the categories developed up to that point. In the later transcripts, new concepts and relationships between categories emerged less frequently, and this pattern was confirmed through analysis of the remaining transcripts. After all transcripts had been analyzed, no additional concepts, subcategories, or relationships between categories emerged. On this basis, the research team judged that theoretical saturation had been reached and concluded the analysis.

Ethical considerations

This study was approved by the Ethics Committee for Clinical Research of the University of the Ryukyus (Approval No. 23-2236-00-00-00; granted January 17, 2024) and was conducted in accordance with the Declaration of Helsinki and Japan's Ethical Guidelines for Medical and Health Research Involving Human Subjects. Written informed consent was obtained from all participants, who were informed of the study's purpose and methods, the voluntary nature of participation, their right to withdraw at any time without disadvantage, and the protection of their personal data. No directly identifying information (e.g., name, address, or medical record number) was collected. Interview recordings and transcripts were anonymized using participant identification codes, and the correspondence table linking these codes to participants' identities was managed separately by a co-investigator on a computer without internet access. Data were stored on an external storage device kept in a locked cabinet in the principal investigator's office, accessible only to the research team, and will be retained for at least five years after the study's completion report or three years after the final publication of its results, whichever is later, after which they will be permanently deleted. Participants who experienced distress during the interview had access to follow-up support through the collaborating hospital.

## Results

Participant characteristics

Of the 11 parent-child pairs enrolled, 10 screened positive on the GADIS-P and were retained in the qualitative analytic sample, while one screened negative and was excluded. The resulting sample of 10 screening-positive parents comprised nine mothers and one father. Because this study's analytic focus was mothers' day-to-day experience of responding to their child and their relationship with the child, and because the participating father described limited day-to-day involvement with his child, his interview was not included in the qualitative analysis presented here. The remaining nine mothers were retained as the final analytic sample. Participants were relabeled A-I to preserve anonymity. Interviews lasted a mean of 38 minutes (median 37 minutes; range 25-54 minutes).

All nine participating parents were mothers, and most were in their 40s (n = 6, 67%). The majority were married (n = 7, 78%), had education beyond high school (n = 6, 67%), and were employed full-time (n = 6, 67%). The median number of cohabiting children per family was three (range 1-6). Of the nine children, five (56%) were male and four (44%) were female, ranging in age from 13 to 15 years (median 14). All nine children had at least one clinical diagnosis, and eight (89%) had two or more co-occurring diagnoses. The most common diagnoses were neurodevelopmental conditions, namely autism spectrum disorder (n = 4, 44%) and attention-deficit/hyperactivity disorder (n = 3, 33%). Sleep-related diagnoses, most often insomnia, were also common (n = 6, 67%). Other mental health conditions, specifically depression, adjustment disorder, mixed anxiety and depressive disorder, oppositional defiant disorder, and social anxiety disorder, were present in six children (67%). Because children could have more than one diagnosis, the percentages presented for diagnostic categories are not mutually exclusive. Participant characteristics and sociodemographic information are summarized in Table 1.

**Table 1:** Sociodemographic and clinical characteristics of participating mothers and their children (N = 9)

Characteristic	n (%)
Mother age group	
30s	2 (22)
40s	6 (67)
50s	1 (11)
Marital status	
Married	7 (78)
Divorced	2 (22)
Education	
High school	3 (33)
Beyond high school	6 (67)
Employment	
Full-time	6 (67)
Part-time	3 (33)
Child sex	
Male	5 (56)
Female	4 (44)
Child age (years)	
13	2 (22)
14	5 (56)
15	2 (22)
Clinical diagnoses of the child	
Two or more diagnoses	8 (89)
Autism spectrum disorder (ASD)	4 (44)
Attention-deficit/hyperactivity disorder (ADHD)	3 (33)
Sleep-related diagnoses	6 (67)
Depression	2 (22)
Adjustment disorder	1 (11)
Mixed anxiety and depressive disorder	1 (11)
Oppositional defiant disorder	1 (11)
Social anxiety disorder	1 (11)

Constant comparative analysis of interview transcripts generated 20 concepts, which were grouped into nine subcategories and, in turn, four categories (Table 2, Figure 1). The resulting analytic structure reflects the range of distinct experiences described across the interviews, rather than an attempt to maximize the number of concepts generated.

**Table 2:** Categories, Subcategories, and Concepts Describing Mothers' Recognition of and Response to Suspected Gaming Disorder

Category	Subcategory	Concept
I. Start of using a game/smartphone and transition to unrestricted use	Necessity and personal ownership of smartphones	Smartphone ownership permitted upon starting middle school
		School-issued tablets for homework
		Restricted outings during the COVID-19 pandemic
	Uncontrolled game/smartphone use	Initial rules giving way to unrestricted use
		All-day device use
II. Manifestation of problems and maternal concerns	Chronically absent from school	Late-night use disrupting morning wake-up
		School non-attendance
	Mental instability	Self-harm or expressions of suicidal ideation
		Angry outbursts and defiance
	Concerns for their future	Excessive in-game spending
		Declining academic performance
	Trouble on social media	Absence of offline friendships
		Concern over contact with online strangers
III. Mental fatigue of mothers and reaching professional support	Isolated child-rearing for mothers	Husband's lack of cooperation
		Inability to rely on extended family
		Mother's own reliance on gaming
	Reaching professional support	Crisis or the child's own request as a turning point
		Navigation of multiple sources of support and personal limits en route to care
IV. Change of parent-child relationship	Changes in parental awareness and behavior	Change in the husband's awareness
		Parental restraint in communication with the child

**Figure 1:**
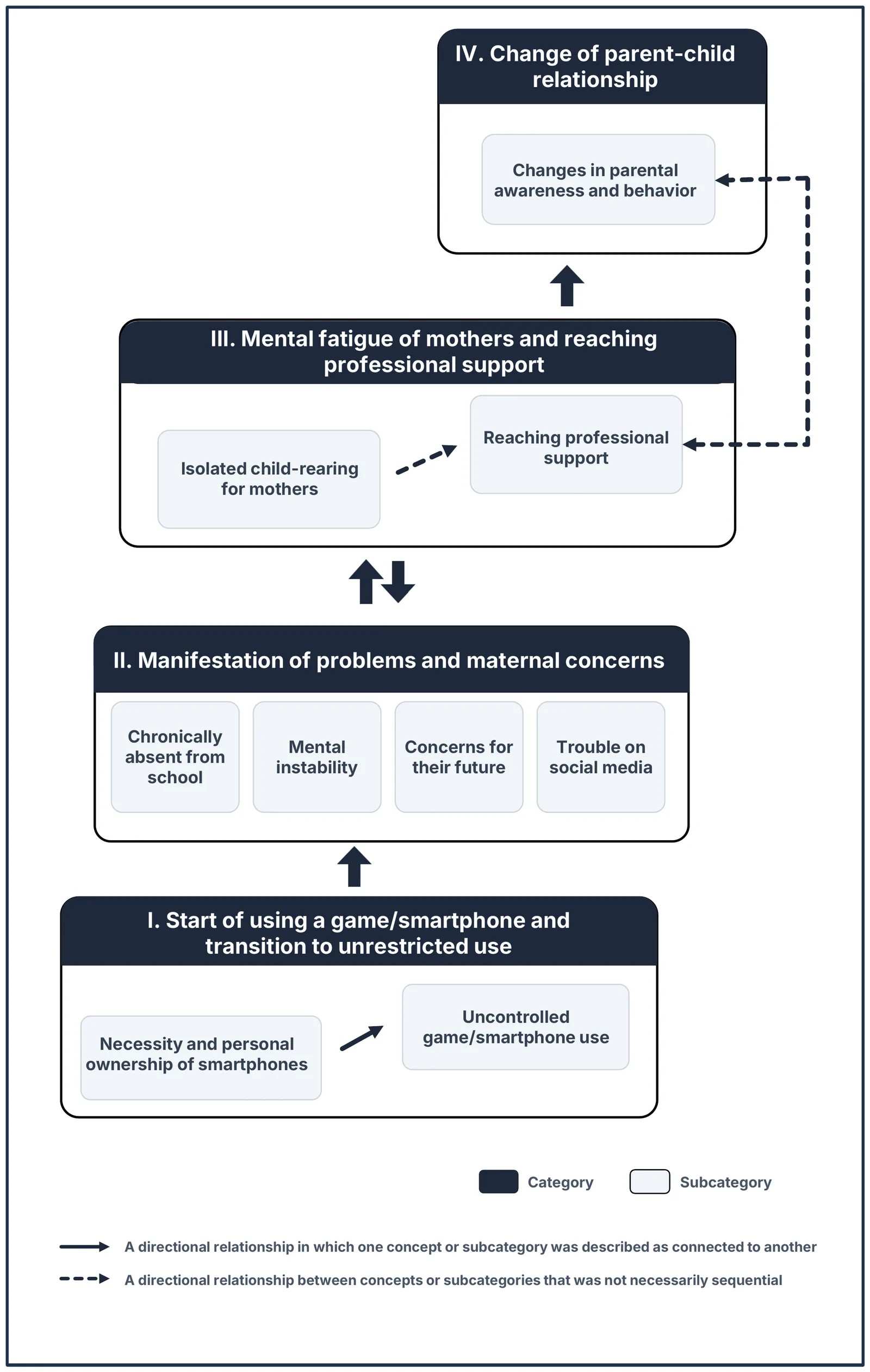
The process of maternal recognition of and response to suspected gaming disorder among Japanese adolescents receiving psychiatric outpatient care. This is an original analytical diagram created by the authors using Microsoft PowerPoint (Redmond, WA, USA) based on the Modified Grounded Theory Approach (M-GTA) analysis presented in this study. The model is based on mothers' retrospectively reconstructed accounts and does not represent a fixed temporal or causal sequence.

Storyline

Mothers' accounts traced a process that began with the "necessity and personal ownership of smartphones" and gave way to "uncontrolled game/smartphone use." As household rules went unenforced, mothers came to recognize increasingly serious manifestations of their child's problems, including being "chronically absent from school," experiencing "mental instability," raising "concerns for their future," and encountering "trouble on social media." Mothers described confronting these escalating problems largely on their own, a pattern of "isolated child-rearing for mothers" that eventually gave way to "reaching professional support," often prompted by a crisis or by the child's own request, though the path to care was rarely direct. Mothers also described "changes in parental awareness and behavior" in some families, as fathers became more actively involved and parents learned to moderate how much they said to their child.

Category I: Start of Using a Game/Smartphone and Transition to Unrestricted Use

Mothers came to allow their child to have a smartphone or gaming device in response to external circumstances, such as school entry or restrictions on going outside during the COVID-19 pandemic, rather than the child's own request. Household rules established at the outset gradually went unenforced, and the child's use became unrestricted.

Mothers illustrated this pattern with specific examples of how their child first acquired a device:

*“Once you're in middle school, you have to have a phone for the school environment*, *for the contact network and [social media platform], *​​​*so that's when he started having one.”* (Participant E)

*“There was quite a lot of tablet homework* (from a school-issued tablet)*, so once he started middle school, I honestly couldn't manage it anymore.”* (Participant B)

*“Because he couldn't go out during COVID, we thought about introducing [a gaming console]* *as a way to let off steam through exercise, so that's how he started using it.”* (Participant E)

However, mothers soon faced uncontrolled game/smartphone use. Mothers described how the rules they had set early on went unenforced as children's use extended across nearly all waking hours on days without school:

*“There were rules at first*, *things like 'you can't play a game past a certain time.' But the rules weren't followed.”* (Participant A)

*“On days off, he has it in his hands constantly from the morning*, *he's on it right up until he goes to sleep.”* (Participant E)

Category II: Manifestation of Problems and Maternal Concerns

Mothers came to recognize increasingly serious manifestations of their child's gaming- and smartphone-related problems, spanning school non-attendance, emotional and behavioral instability, worries about the child's future, and difficulties on social media.

Mothers described a pattern in which nighttime device use extended progressively later, disrupting sleep, and described this culminating in their child's chronic school absence or withdrawal:

*“He doesn't sleep at night, staying up until morning. His body isn't moving, so his brain doesn't get tired until late*, *that's abnormal, when you think about it.”* (Participant H)

*“He eventually stopped going to school around the end of that first year.”* (Participant F)

Mothers also described their child's mental instability, including direct self-harm, such as searching for a knife or cutting, and suicidal ideation expressed during a crisis:

*“He looks for a knife, and there's been some light cutting too.”* (Participant B)

*“She ran away and shut herself in a bathroom... in the messages back and forth, she actually told me she wanted to die.” *(Participant C) (Similar experiences of self-harm or suicidal ideation were also described by Participants D and G.)

Mothers also described this instability surfacing as anger and irritability during gameplay, in children who, upon losing or not getting their way, would lash out and sometimes damage property:

*“He kicked the fan and broke it.”* (Participant I)

Mothers also expressed concern about their child's future, citing excessive in-game spending and declining academic performance:

*“Over about four months, unauthorized charges came to around 100,000 yen.”* (Participant H)

*“Out of 150 students, she's at the very bottom*, *first or second from last.”* (Participant G)

Finally, mothers described trouble on social media, including children with few offline relationships and concern about strangers encountered online:

*“She doesn't really have friends outside of the internet.”* (Participant C)

*“I don't know what kind of people she's connecting with on [social media platforms], I worry she could be deceived.”* (Participant G)

Category III: Mental Fatigue of Mothers and Reaching Professional Support

Mothers experienced mounting mental fatigue as they bore the day-to-day burden of responding to their child's problem behaviors, often feeling completely alone in doing so.

Mothers described minimal cooperation from their husbands in managing the situation and no support from extended family, reflecting isolated child-rearing:

*“I can't really expect much from my husband.”* (Participant F)

*“We can't really rely on my husband's parents, and my own parents live outside the prefecture, so it's really just up to my husband and me.”* (Participant I)

Facing this isolation and exhaustion, mothers described turning to gaming or other coping behaviors themselves, as a way to unwind:

*“I wonder if it's that we parents end up relying on these game devices ourselves.”* (Participant C)

*“I play games too, on my phone. I have a drink, then play a bit, and go to sleep.”* (Participant G)

Mothers described this isolation and exhaustion eventually giving way to reaching professional support, though the path to care was rarely direct; for some, a specific crisis or the child's own request marked the turning point:

*“She shut herself in a bathroom, and we called the police…in our messages, she told me she wanted to die, so I thought, let's go to the hospital.”* (Participant C)

*“At that point, she herself said she wanted to go to the hospital and try counseling.”* (Participant D)

*“He had always refused, saying he'd manage on his own. But then there'd be moments where it would just explode and become unmanageable.” *(Participant I)

Mothers also described reaching care as involving recognizing their own limits or navigating multiple sources of support:

*“I suddenly burst into tears at that comment, and that's when I finally accepted that I was at my limit.”* (Participant B)

Category IV: Change of Parent-Child Relationship

Mothers described gradually shifting their own awareness and approach toward their child as they worked through the child's gaming-related problems. Fathers became more actively involved, and parents learned to moderate how much they said to their child.

Mothers described a shift in their husbands' engagement once the family began seeking outside support:

*“Because of that* (seeking outside support)*, my husband started getting involved too*, *I really think his awareness has changed.”* (Participant B)

Mothers also described consciously scaling back how much they said to their child, following guidance to avoid escalating conflict:

*“I've stopped raising my voice about it so much. I only bring up the bare minimum, the truly dangerous things, like money or strangers. Otherwise, I don't push hard anymore.”* (Participant G)

## Discussion

This exploratory, interview-based study examined how mothers of Japanese adolescents with suspected GD recognized and responded to their child's gaming- and smartphone-related problem behaviors. Mothers' accounts traced a process that began with the acquisition of a smartphone or gaming device and, through eroding household rules and escalating problem behaviors, culminated in maternal exhaustion; some families also experienced changes in parental roles. Previous qualitative studies have primarily examined adolescents' own accounts of gaming problems and treatment (e.g., Gurdal et al. [24]; Bore et al. [25]), whereas research centering on parents remains limited. To the best of our knowledge, Werner et al. [17] is the closest prior study, examining Swedish parents' perceived need for support after their children had received treatment. That study found that parents, while motivated by a strong sense of purpose, often struggled to manage their child's gaming and sought both practical guidance and relational support from partners and other parents facing similar challenges, findings that resonate with the isolation and support-seeking described by mothers in the present study. Furthermore, all participating children in the present study had at least one developmental or mental health diagnosis alongside their suspected GD, representing a clinically complex population that is rarely the explicit focus of parent-perspective research. The present study therefore extends this limited literature by providing in-depth insight into how mothers experienced and responded to their child's suspected GD within this complex clinical context.

The trajectory described in Category I speaks to a persistent ambiguity in the family-factors literature regarding household rules. In the context of our findings, "uncontrolled use" refers to the erosion of household rules and the resulting unrestricted gaming and smartphone use. Petrescu et al.'s [11] systematic review of 64 studies found that, unlike parental knowledge, which was identified as a protective factor, restrictive mediation produced inconclusive results; the longitudinal studies included in that review pointed to a bidirectional relationship, with parental rule-setting sometimes tightening and sometimes loosening as children's gaming symptoms changed over time. The present findings suggest one explanation for this ambiguity: mothers described rules that were established early, often around the time their child started middle school or received a personal device, but that were not sustained as gaming became more central to daily life. A cross-sectional measure of "having a rule" may therefore miss this within-family shift from initial restriction to eventual non-enforcement.

Category II encompassed a markedly heterogeneous set of concerns, ranging from academic decline and in-game spending to school non-attendance, self-harm, and suicidal ideation. This breadth is consistent with the wide-ranging functional impairment described in the diagnostic conceptualization of GD, which extends beyond gaming behavior itself to encompass personal, family, social, educational, and occupational domains [1]. This breadth mirrors findings from prior research. Among Japanese students, GD has been associated with school avoidance or absenteeism and with greater spending on in-game purchases [26]; among adolescent gamers more broadly, it has been associated with a higher likelihood of self-harm and suicidal ideation [27]. However, all nine children in this study had at least one documented clinical diagnosis in addition to suspected GD, and eight had two or more such co-occurring diagnoses, most commonly autism spectrum disorder or attention-deficit/hyperactivity disorder. Some of the concerns mothers described may therefore reflect these underlying conditions as much as gaming-specific processes: academic difficulties have been linked to attention-deficit/hyperactivity disorder [28], peer relationship difficulties to attention-deficit/hyperactivity disorder [29], and emotional regulation difficulties to autism spectrum disorder [30], while anger and defiance are core features of oppositional defiant disorder or attention-deficit/hyperactivity disorder-related impulsivity. Self-harm and suicidal ideation, however, were described across children with a range of different diagnoses rather than any single condition, and given the extensive diagnostic overlap in this sample, individual concerns cannot be attributed with certainty to any one underlying condition. Some children in this sample also had documented sleep-related diagnoses, which may be relevant to broader concerns such as school non-attendance. These accounts, therefore, are best read as reflecting circumstances where gaming-related problems and other developmental or mental health vulnerabilities frequently coexisted, rather than as suggesting that gaming was their sole cause.

The family-factors literature has focused mainly on how family functioning shapes the child's gaming outcomes [12,13]. Category III shifts that focus, pointing instead to the toll that managing a child's gaming-related problems takes on the parent. In this study, that parent was the mother, who served as the family's primary respondent to these problems. By their own accounts, these mothers frequently managed the problem largely alone, describing minimal practical or emotional support from their husbands and an absence of extended-family support as well. This scarcity of spousal and extended-family support, and the resulting concentration of the burden on mothers, echoes the broader theme of family disharmony identified as a risk factor for adolescent GD in recent reviews [11]. In Japan, long paternal working hours are common and structurally limit fathers' time for childcare [31], yet reduced working hours alone do not close the gap. A large Japanese national cohort study has shown that fathers' limited involvement in childcare and housework persists even among those working comparatively short hours, a pattern attributed to entrenched gender role norms rather than time constraints alone [32]. This suggests that the burden placed on mothers may reflect longstanding family role divisions, that is, the persistent concentration of childcare and household responsibilities on mothers, even when fathers' working hours are relatively short, rather than a response specific to the child's gaming-related problems. For these mothers, this exhaustion did not resolve on its own but instead prompted contact with professional support. For some, this occurred abruptly, triggered by an acute crisis such as self-harm or a marked behavioral escalation; for others, it followed a longer process of recognizing their own limits or navigating several potential sources of help before eventually reaching the hospital.

Category IV, reflecting a shift in parental awareness or approach, captured a pattern identified within the interview data. Reaching professional support (Category III) did not by itself lead to this kind of change; rather, the underlying isolation and caregiving burden often persisted. Where change did occur, mothers' narratives placed it around the time the family began seeking outside support. The changes described, greater paternal involvement and parents moderating how much they said to their child, suggest that seeking that support offered some families an occasion to reconsider family roles and approaches to their child that had previously gone unexamined. This is consistent with a qualitative study of practitioners treating adolescent internet addiction, which found that conjoint therapy sessions involving family members fostered effective communication and helped restore family relationships and functioning, although practitioners also emphasized that the appropriateness and degree of family involvement needed to be judged on a case-by-case basis [33]. Qualitative accounts from adolescents themselves similarly describe reduced family conflict and improved mutual understanding with parents following psychological treatment for gaming-related problems [24,25]. However, the present study's single-interview design cannot establish whether this shift preceded, followed, or coincided with help-seeking.

These findings suggest several implications for clinical practice. First, clinical assessment of families of adolescents with suspected GD may benefit from attending not only to the child's symptoms and gaming duration but also to household rules and family responses to gaming-related problems. Second, the isolation and mental fatigue described by mothers suggest that clinical assessment may also benefit from attention to mothers' psychological burden and family role divisions, rather than focusing solely on the child's gaming-related problems. Third, at initial contact, clinicians may benefit from assessing not only gaming-related problems but also their impact on family life and the parent-child relationship, as well as the family's previous responses and support needs.

Strengths and limitations

This study has several strengths. By focusing on mothers' own accounts, it addresses a perspective that remains underrepresented in the literature on adolescent gaming problems, and by capturing the process from mothers' initial recognition of their child's difficulties through their pursuit of professional support, it extends the perspective beyond the post-treatment experiences examined in some prior studies. The clinically complex nature of the sample, in which all participating children had at least one co-occurring developmental or mental health diagnosis, adds to the limited literature on mother perspectives in clinically complex families. Methodologically, the use of M-GTA enabled constant comparative analysis of similarities and differences among participants, including attention to disconfirming instances, and allowed changes in mothers' recognition and response to be understood in relation to parent-child interaction and the pursuit of support.

This study also has several limitations. First, participants were a convenience sample recruited from a single specialized psychiatric hospital, and the qualitative analysis was restricted to mothers whose children screened positive on the GADIS-P. Thus, the findings do not capture how mothers recognize and respond to gaming-related difficulties before their children enter psychiatric care. The transferability of the process model beyond this clinical population may therefore be limited. Second, all the children had a developmental or mental health diagnosis; the extensive diagnostic overlap in this sample limits the extent to which the process described can be attributed specifically to suspected GD, as opposed to these co-occurring conditions. Third, because data were collected through a single retrospective interview, the process described in the storyline reflects mothers' retrospective accounts of these experiences. These accounts, particularly regarding sensitive or high-impact events, may be subject to recall bias or retrospective reinterpretation, and the study design limits inference regarding the temporal ordering or causality of the process described.

Future research should use longitudinal designs to examine how mothers' recognition of gaming-related problems, household responses, and help-seeking change over time, and to identify turning points and conditions that shape this process. Future studies should also include fathers, grandparents, and other caregivers, in addition to mothers, to examine how experiences may differ across family roles and relationships with the child. Intervention research should further examine the feasibility and acceptability of family-based approaches and evaluate their effects on family and adolescent outcomes.

## Conclusions

This study described the process by which mothers of Japanese adolescents with suspected GD receiving psychiatric outpatient care recognized and responded to their child's gaming- and smartphone-related problem behaviors. As household rules eroded, concerns escalated to include school non-attendance, emotional instability, worries about the child's future, and difficulties on social media. For mothers, this process involved mounting isolation and fatigue; in some families, it also coincided with a shift in parental roles, while caregiving needs often remained ongoing. These findings suggest that clinical assessment of families of adolescents with suspected GD may benefit from attending to mothers' own experiences alongside the child's symptoms. Mothers' isolation and fatigue were described as ongoing experiences rather than being resolved at a single point in time. Continued contact with these families may therefore be worth considering.

## Appendices

Interview guide

English translation of the interview guide used in the study.

Section 1: Basic Information Q1. Number of cohabiting children Response Options: 1 / 2 / 3 / 4 or more Q2. Child's age (school grade) Response Options: 4th grade elementary (age 10+) / 5th grade elementary / 6th grade elementary / 1st year junior high / 2nd year junior high / 3rd year junior high / 1st year high school / 2nd year high school / 3rd year high school (age 17) Q3. Whether the child plays internet-connected games (e.g., smartphone, Nintendo Switch, PlayStation, PC) Response Options: Plays internet games / Does not play internet games → survey ends Q4. Place of residence Response Options: Naha City / Urasoe City / Other (____) City Q5. Age group Response Options: 20s / 30s / 40s / 50s / 60s or older Q6. Sex Response Options: Male / Female / Other Q7. Occupation Response Options: Part-time / temporary employee / dispatched or contract employee / full-time employee / self-employed or business owner / other Q8. Education Response Options: High school graduate / junior college / vocational school / university / graduate school / other Q9. Household annual income Response Options: Under 2 million yen / 2-4 million yen / 4-6 million yen / 6-8 million yen / 8-10 million yen / 10-12 million yen / 12 million yen or more Q10. Cohabiting family members Response Options: Spouse / children / grandchildren / own parents / spouse's parents / other

Section 2: Questions About Gaming Q1. When did your child start playing internet games? Q2. What game title(s) has your child been absorbed in over the past year? Q3. Has there been excessive in-game spending over the past year? Q4. Are there any family-established rules regarding gaming? If so, what specifically are they? Q5. (Asked only if a household rule exists in Q4) Does your child follow the household rules regarding gaming? Q6. Do you use any parental monitoring/control functions? Q7. What gaming device does your child mainly use? Q8. Approximately how much time does your child spend gaming on weekdays? Q9. Approximately how much time does your child spend gaming on days off? Q10. Approximately how many days per week does your child play games? Q11. What gaming-related problem behavior are you most concerned about? Q12. Please share your (the parents') thoughts on gaming in general. Q13. Do you have any concerns regarding your child's friendships? Q14. Do you have any concerns regarding the parent-child relationship? Q15. Is there anything else concerning about your child that you would like to share?
 
